# Erysipelas1Read at the thirty-second annual meeting of the Medical Association of Central New York, at Syracuse, October 17, 1899.

**Published:** 1900-06

**Authors:** A. A. Young

**Affiliations:** Newark, N. Y.


### ERYSIPELAS.[1]

BY A. A. YOUNG, M.D., Newark, N. Y.

ERYSIPELAS may be defined, so far as the ocular manifestations are concerned, as an intense specific dermatitis, an inflammation in which the lymph channels are largely, if not entirely involved.

That this disease is induced by the deposition and growth of specific microörganisms, apparently the streptococcus pyogenes, can scarcely be doubted. The disease is certainly contagious, and as

1. Read at the thirty-second annual meeting of the Medical Association of Central New York, at Syracuse, October 17, 1899.

such the inducing agent must have reproductive powers which are capable of self-growth and self-development. Microörganisms, like other organisms, do not spring into being from nothing, full grown, there must be a germinal vesicle however small; there must be a period of growth however short.

What is true of the development of organic life in the macroscopic world must be inferentially true in the microscopic world; what is so apparent in the development of insect life must be practically true in the development of other forms of life, though not so apparent to the senses. The streptococcus can form no exception to the rule, it must have its beginning, it must have its active period of development, reaching to and ending in perfectness before it accomplishes its biological cycle and is prepared for the process of reproduction of its species. It may require but an hour for it to complete, under favorable conditions, its biological cycle, but that short period does not affect the process. Its embryonic life, though short, must exist.

I have said that erysipelas is a violent inflammation of the lymph channels, but that is only the ocular manifestations of it,—its local phase. In and about the lymph channels specific streptococci may be found in abundance, but it is doubtful if their development occurs at the point of inflammation. The inflammatory patch is apparently the nidus of the fully developed coccus, while the developing coccus occupies other portions of the body making of the affection a systemic rather than a local one.

In true cutaneous erysipelas there are premonitory symptoms consisting of a chill, fever and general malaise in a variable degree prior to the appearance of the eruption. This is a marked indication that the affection is a systemic one and that the germ develops elsewhere than in the lymph channels, and occupying the lymph channels only after development has been completed. There is a similarity of method between the development of erysipelas and the eruptive fevers, sufficient similarity that if the latter be constitutional affections with local manifestations then erysipelas must be so classed also.

It is of but little moment whether a case of erysipelas be idiopathic or traumatic, the inducing agent must be a like coccus, having like powers and like methods of development; and its successful treatment must be accomplished by similar methods and means. Under our present knowledge, then, the division of erysipelas into idiopathic and traumatic can readily be dispensed with, for it only shows a method of entrance and not a method of development; how a germ enters is comparatively valueless to how this same germ may be destroyed and eliminated, it is therefore the province of medicine to accomplish the latter as immunisation is, as yet out of the question. It is still questionable whether erysipelas is transmissible directly from parent to child or not; there are some conditions at least that indicate the truth of the assumption that it is so transmissible. That the disease may begin in the individual child and continue through life appearing at intervals more or less frequent is a common observation.

If in the child there be recurrent attacks coming apparently from unknown and undiscoverable sources; if the disease be a constitutional one and not an independent and isolated dermatitis, then it is easy to infer of the transmissibility of the affection from parent to child; if it be not hereditary then it must be admitted that abnormal conditions or weakness are transmitted to the child, which make the entrance and development of the specific germ comparatively easy, whether such development be in the skin, the cellular and intermediate tissues or in some other and more remote medium of the body—this medium I believe to be the blood itself.

The entrance of the coccus, as a spore or as a fully developed organism, may be by way of the skin and the inflamed area may enclose the point through which it entered, but this act alone does not constitute the disease—there must be germ development beginning with or even prior to the febrile manifestations; there must be a period of incubation, a period of development and a period of perfectness, short though these periods may be. Inferentially the developing coccus is in the blood stream, active and growing; an activity that produces the febrile disturbances and general malaise, which precede the characteristic eruption, and eruption marking the home of the fully developed coccus in which home it prepares itself for the office of reproduction by depositing its germinal vesicles which can be readily absorbed into the blood stream, forming a new or reinfection.

Immediately with and following upon germ invasion, phagocytes increase and multiply in number and in proportion to this increase together with the amount of vitality which they possess, is the severity of the disease lessened; increased phagocytosis means decreased morbid conditions.

Concerning the three phases or conditions of erysipelas, viz.: the cutaneous, cellulocutaneous and cellular, I do not care at this time to consider for they are doubtless of the same variety, produced by a like coccus though treatment must be modified considerably for each existing condition; the treatment for the former being almost exclusively medical, while for the latter medico-surgical.

Regarding the disease as a systemic one and of microbic origin, and from which conclusion legitimately the inference is drawn that the coccus develops within the blood or other circulatng fluid of the body, then two lines of treatment are suggested, both tending to the same end, viz.: the destruction and elimination of the specific coccus. There must then be an internal medication, systemic in character, one which will modify and devitalise the developing coccus. Nature has already furnished such an agent in the phagocyte, the multiplicity and activity of which ought to be encouraged. For this purpose one of the most satisfactory preparations that has come under my observation is nuclein, to its revitalising properties the system most quickly responds, as a stimulant to phagocytosis its value is most apparent.

In accordance with custom, quinine and iron, preferably the sulphate, on account of its greater antiseptic properties, have been prescribed and with apparent good results. It appears that the benefit to be derived from these agents comes largely from their germicidal properties, checking the growth and perhaps destroying the semi-developed coccus by contact.

Since by theory the inflamed area is the nidus of the fully developed coccus, a theory applicable to all specific eruptive fevers, most important, then, is external medication; by the use of germicides we are able to modify and even destroy the coccus before it begins its process of reproduction which means systemic reinfection. To destroy the coccus in situ must be the aim of local medication.

In passing, allow me to add that the use of the poultice or other hot fomentation, recommended in our text-books frequently, I most unqualifiedly condemn, it is a useless non-aseptic and nasty appliance; it is doubtless a germ developer instead of a germ destroyer.

The medicinal agents which have served my purpose best are aconite and sulphate of iron, applied over the entire inflamed area. Formerly the tincture of aconite was used with the iron, but it made of the patient an unsightly being, though these agents were effective in their action. During later years aconitia has been substituted for the tincture of aconite with equally good results and thus escaping the disagreeable color produced by the latter.

It is doubtful if aconite possesss any direct germicidal properties, but its sedative properties are marked, and by virtue of which it lessens in extent and severity inflammatory action by rendering inactive the agents that produce it; time is thus allowed for their destruction and expulsion by other methods and agencies.

As a germicide and one specially fitted for this specific coccus, and many have been tried, I have found nothing that equals the sulphate of iron in efficiency, its activity seems to be increased however by combining it with some preparation of aconite preferably aconitia; from this combination I have obtained none other than satisfactory results.

In the administration of these drugs only one object is sought for viz.: the destruction of the coccus in its nidus, in the inflamed dermal patch. This work these agents do well.

Since erysipelas in a certain degree is a specific eruptive fever and corresponds to the type of such fevers, as might be expected, we have exfoliation of the affected skin; then as a precautionary measure a solution of formaldehyde for bathing purposes daily is advisable. In the cellular variety usually the aid of the surgeon must be invoked, and the débris allowed to escape early, after which antiseptic dressings must be applied and free drainage continued so long as active streptococci remain. This wound forms a means of exit, not for the local, fully developed cocci alone, but for the developing ones found elsewhere as well.

In these surgical cases strict asepsis and strict cleanliness are imperative; the adage here applies with peculiar force that "Godliness is next to cleanliness."

22 E. Miller Street.

